# C-type Nd_2_Se_3_

**DOI:** 10.1107/S1600536809005455

**Published:** 2009-02-21

**Authors:** Christof Schneck, Patrick Höss, Thomas Schleid

**Affiliations:** a Institut für Anorganische Chemie, Universität Stuttgart, Pfaffenwaldring 55, D-70569 Stuttgart, Germany

## Abstract

The title compound, neodymium sesquiselenide, is isotypic with the other known rare-earth metal(III) selenides *M*_2_Se_3_ (*M* = La–Pr and Sm–Lu) with the cubic C-type structure. It adopts a cation-defective Th_3_P_4_-type arrangement with close to 8/9 of the unique neodymium-cation site occupied, leading to the composition Nd_2.667_Se_4_ (*Z* = 4) or Nd_2_Se_3_ (*Z* = 5.333), respectively. The Nd^3+^ cations are thus surrounded by eight selenide anions, forming trigonal [NdSe_8_]^13−^ dodeca­hedra, whereas the Se^2−^ anions exhibit a sixfold coordination, but due to the under-occupation of neodymium, each one is statistically surrounded by only 5.333 cations. The crystal studied was a merohedral twin with a 0.31 (6):0.69 (6) domain ratio.

## Related literature

For the structural family with the cation-defective Th_3_P_4_-type arrangement, see: Pardo *et al.* (1963 ▶); Flahaut *et al.* (1965 ▶); Lashkarev & Paderno (1965 ▶). For the rare-earth sesquiselen­ides *M*_2_Se_3_ with *M* = La–Pr and Sm–Lu, see: Grundmeier & Urland (1995 ▶); Folchnandt (1997 ▶); Folchnandt & Schleid (2001 ▶); Folchnandt *et al.* (2004 ▶).

## Experimental

### 

#### Crystal data

Nd_2.667_Se_4_
*M* *_r_* = 700.48
Cubic, 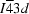

*a* = 8.8632 (6) Å
*V* = 696.26 (8) Å^3^
*Z* = 4
Mo *K*α radiation
μ = 40.39 mm^−1^
*T* = 293 K
0.03 × 0.03 × 0.02 mm

#### Data collection

Stoe IPDS-I diffractometer
Absorption correction: numerical (*X-SHAPE*; Stoe & Cie, 1999 ▶) *T*_min_ = 0.305, *T*_max_ = 0.401
8964 measured reflections
220 independent reflections
214 reflections with *I* > 2σ(*I*)
*R*_int_ = 0.065

#### Refinement

*R*[*F* ^2^ > 2σ(*F* ^2^)] = 0.026
*wR*(*F* ^2^) = 0.060
*S* = 1.22
220 reflections
7 parameters
Δρ_max_ = 1.01 e Å^−3^
Δρ_min_ = −2.11 e Å^−3^
Absolute structure: Flack (1983 ▶), 92 Friedel pairs
Flack parameter: 0.31 (6)

### 

Data collection: *DIF4* (Stoe & Cie, 1992 ▶); cell refinement: *DIF4*; data reduction: *REDU4* (Stoe & Cie, 1992 ▶); program(s) used to solve structure: *SHELXS97* (Sheldrick, 2008 ▶); program(s) used to refine structure: *SHELXL97* (Sheldrick, 2008 ▶); molecular graphics: *DIAMOND* (Brandenburg, 2006 ▶); software used to prepare material for publication: *SHELXL97*.

## Supplementary Material

Crystal structure: contains datablocks I, global. DOI: 10.1107/S1600536809005455/mg2064sup1.cif

Structure factors: contains datablocks I. DOI: 10.1107/S1600536809005455/mg2064Isup2.hkl

Additional supplementary materials:  crystallographic information; 3D view; checkCIF report

## Figures and Tables

**Table 1:** Selected bond lengths (Å)

Nd—Se^i^ (4×)	2.9675 (5)
Nd—Se (4×)	3.1732 (6)

Symmetry codes: (i) 
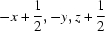
.

**Fig. 1.**
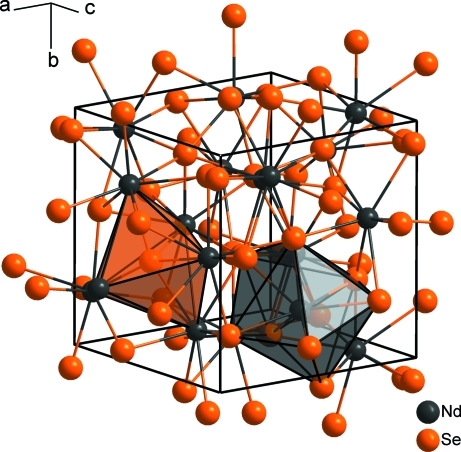
Crystal structure of C-Type Nd2Se3.

**Fig. 2.**
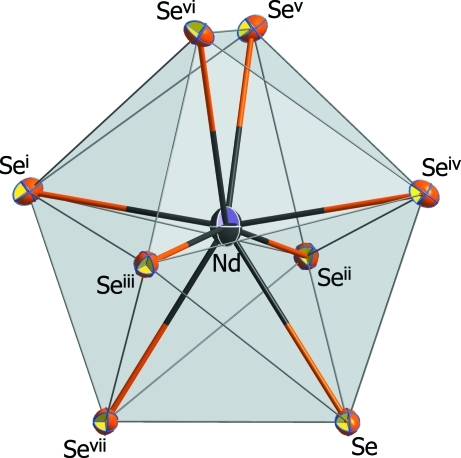
Coordination sphere of the Nd3+ cation in the shape of a trigonal dodecahedron. Displacement ellipsoids are drawn at 95% probability level. [Symmetry codes: (i) -x + 1/2, -y, z + 1/2; (ii) y + 1/4, x + 1/4, z + 1/4; (iii) y + 1/4, -x - 1/4, -z + 1/4; (iv) -x + 1/2, y, -z; (v) -y + 3/4, -x + 1/4, z + 1/4; (vi) -y + 3/4, x - 1/4, -z + 1/4; (vii) x, -y, -z + 1/2.]

**Fig. 3.**
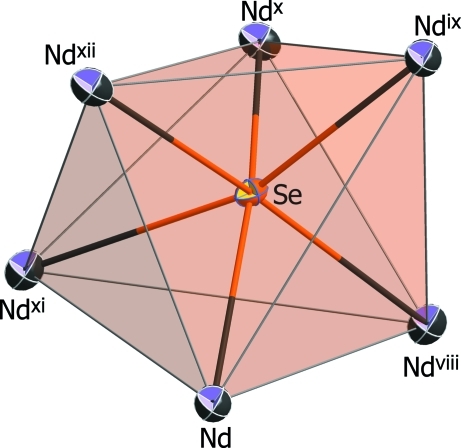
Coordination sphere of the Se2- anion. Due to the under-occupation of the neodymium site, each selenium is surrounded by 5.333 Nd3+ cations. Displacement ellipsoids are drawn at 95% probability level. [Symmetry codes: (viii) y, -z, -x + 1/2; (ix) -x + 1/2, -y, z - 1/2; (x) -y - 1/4, x - 1/4, -z + 1/4; (xi) y, z, x; (xii) y + 1/4, -x + 3/4, -z + 1/4.]

## supplementary crystallographic information

### Comment

C-type Nd_2_Se_3_ (Fig. 1) belongs to a structural family with the cation-defect Th_3_P_4_-type arrangement (Pardo *et al.*, 1963; Flahaut *et al.*, 1965; Lashkarev & Paderno, 1965) adopted by rare-earth sesquiselenides *M*_2_Se_3_ with *M* = La – Pr and Sm – Lu (Grundmeier & Urland, 1995; Folchnandt, 1997; Folchnandt & Schleid, 2001; Folchnandt *et al.*, 2004) following the general formula *M*_2.667_□_0.333_Se_4_. The Nd^3+^ cations occupy the 12*a* position, whereas selenium resides at the 16*c* position. Despite the fact that out of the 12 possible cationic sites (per 16 Se^2-^ and unit cell), only 10.667 are allowed to be occupied to realise the composition Nd_2_Se_3_ (with *Z* = 5.333, *i.e. M*_2.667_□_0.333_Se_4_ with *Z* = 4); these exhibit the coordination number 8 with respect to the selenide anions. The [NdSe_8_]^13-^ coordination polyhedra can be described as trigonal dodecahedra with 4-symmetry (Fig. 2). On average, the Se^2-^ anions are surrounded by 5.333 Nd^3+^ cations in a trigonal hemiprism of symmetry .3. with faces rotated 38.2° with respect to each other (Fig. 3).

### Experimental

Ruby-red, multifaceted, transparent crystals of Nd_2_Se_3_ were obtained from stoichiometric reaction of the elements in the presence of CsCl as a flux, placed within a torch-sealed evacuated fused-silica vessel. The mixture was heated at 1123 K for seven days, followed by cooling to ambient temperature with 10 K/h.

### Crystal data

**Table:**

Nd_2.667_Se_4_	*D*_x_ = 6.682 Mg m^−^^3^
*M**_r_* = 700.48	Mo *K*α radiation, λ = 0.71069 Å
Cubic, *I*43*d*	Cell parameters from 5000 reflections
Hall symbol: I -4bd 2c 3	θ = 1.0–32.7°
*a* = 8.8632 (6) Å	µ = 40.39 mm^−^^1^
*V* = 696.26 (8) Å^3^	*T* = 293 K
*Z* = 4	Block, red
*F*(000) = 1184	0.03 × 0.03 × 0.03 mm

### Data collection

**Table:**

Stoe IPDS-I diffractometer	220 independent reflections
Radiation source: fine-focus sealed tube	214 reflections with *I* > 2σ(*I*)
graphite	*R*_int_ = 0.065
imaging plate detector system scans	θ_max_ = 32.7°, θ_min_ = 5.6°
Absorption correction: numerical (*X-SHAPE*; Stoe & Cie, 1999)	*h* = −13→13
*T*_min_ = 0.305, *T*_max_ = 0.401	*k* = −13→13
8964 measured reflections	*l* = −13→13

### Refinement

**Table:**

Refinement on *F*^2^	Secondary atom site location: difference Fourier map
Least-squares matrix: full	*w* = 1/[σ^2^(*F*_o_^2^) + (0.0359*P*)^2^] where *P* = (*F*_o_^2^ + 2*F*_c_^2^)/3
*R*[*F*^2^ > 2σ(*F*^2^)] = 0.026	(Δ/σ)_max_ = 0.007
*wR*(*F*^2^) = 0.060	Δρ_max_ = 1.01 e Å^−^^3^
*S* = 1.22	Δρ_min_ = −2.11 e Å^−^^3^
220 reflections	Extinction correction: *SHELXL97* (Sheldrick, 2008), Fc^*^=kFc[1+0.001xFc^2^λ^3^/sin(2θ)]^-1/4^
7 parameters	Extinction coefficient: 0.0086 (7)
0 restraints	Absolute structure: Flack (1983), 92 Friedel pairs
Primary atom site location: structure-invariant direct methods	Flack parameter: 0.31 (6)

### Special details

**Table:**

Geometry. All e.s.d.'s (except the e.s.d. in the dihedral angle between two l.s. planes) are estimated using the full covariance matrix. The cell e.s.d.'s are taken into account individually in the estimation of e.s.d.'s in distances, angles and torsion angles; correlations between e.s.d.'s in cell parameters are only used when they are defined by crystal symmetry. An approximate (isotropic) treatment of cell e.s.d.'s is used for estimating e.s.d.'s involving l.s. planes.
Refinement. Refinement of *F*^2^ against ALL reflections. The weighted *R*-factor *wR* and goodness of fit *S* are based on *F*^2^, conventional *R*-factors *R* are based on *F*, with *F* set to zero for negative *F*^2^. The threshold expression of *F*^2^ > σ(*F*^2^) is used only for calculating *R*-factors(gt) *etc*. and is not relevant to the choice of reflections for refinement. *R*-factors based on *F*^2^ are statistically about twice as large as those based on *F*, and *R*- factors based on ALL data will be even larger.

### Fractional atomic coordinates and isotropic or equivalent isotropic displacement parameters (Å^2^)

**Table:**

	*x*	*y*	*z*	*U*_iso_*/*U*_eq_	Occ. (<1)
Nd	0.3750	0.0000	0.2500	0.0053 (2)	0.89
Se	0.07261 (5)	0.07261 (5)	0.07261 (5)	0.0028 (3)	

### Atomic displacement parameters (Å^2^)

**Table:**

	*U*^11^	*U*^22^	*U*^33^	*U*^12^	*U*^13^	*U*^23^
Nd	0.0066 (3)	0.0046 (3)	0.0046 (3)	0.000	0.000	0.000
Se	0.0028 (3)	0.0028 (3)	0.0028 (3)	0.00063 (16)	0.00063 (16)	0.00063 (16)

### Geometric parameters (Å, °)

**Table:**

Nd—Se^i^	2.9675 (5)	Nd—Se	3.1732 (6)
Nd—Se^ii^	2.9675 (5)	Se—Nd^viii^	2.9675 (5)
Nd—Se^iii^	2.9675 (5)	Se—Nd^ix^	2.9675 (5)
Nd—Se^iv^	2.9675 (5)	Se—Nd^x^	2.9675 (5)
Nd—Se^v^	3.1732 (6)	Se—Nd^xi^	3.1732 (6)
Nd—Se^vi^	3.1732 (6)	Se—Nd^xii^	3.1732 (6)
Nd—Se^vii^	3.1732 (6)		
			
Se^i^—Nd—Se^ii^	91.403 (3)	Se^ii^—Nd—Se	77.283 (2)
Se^i^—Nd—Se^iii^	91.403 (3)	Se^iii^—Nd—Se	87.467 (16)
Se^ii^—Nd—Se^iii^	162.00 (2)	Se^iv^—Nd—Se	67.092 (10)
Se^i^—Nd—Se^iv^	162.00 (2)	Se^v^—Nd—Se	135.510 (1)
Se^ii^—Nd—Se^iv^	91.403 (3)	Se^vi^—Nd—Se	135.510 (1)
Se^iii^—Nd—Se^iv^	91.403 (3)	Se^vii^—Nd—Se	64.738 (1)
Se^i^—Nd—Se^v^	77.284 (2)	Nd^viii^—Se—Nd^ix^	88.609 (17)
Se^ii^—Nd—Se^v^	67.092 (10)	Nd^viii^—Se—Nd^x^	88.609 (17)
Se^iii^—Nd—Se^v^	130.811 (11)	Nd^ix^—Se—Nd^x^	88.609 (17)
Se^iv^—Nd—Se^v^	87.468 (16)	Nd^viii^—Se—Nd^xi^	107.535 (2)
Se^i^—Nd—Se^vi^	87.468 (16)	Nd^ix^—Se—Nd^xi^	162.372 (6)
Se^ii^—Nd—Se^vi^	130.811 (11)	Nd^x^—Se—Nd^xi^	84.849 (2)
Se^iii^—Nd—Se^vi^	67.092 (10)	Nd^viii^—Se—Nd^xii^	162.372 (6)
Se^iv^—Nd—Se^vi^	77.283 (2)	Nd^ix^—Se—Nd^xii^	84.849 (2)
Se^v^—Nd—Se^vi^	64.739 (1)	Nd^x^—Se—Nd^xii^	107.534 (2)
Se^i^—Nd—Se^vii^	67.092 (10)	Nd^xi^—Se—Nd^xii^	81.565 (16)
Se^ii^—Nd—Se^vii^	87.468 (16)	Nd^viii^—Se—Nd	84.849 (2)
Se^iii^—Nd—Se^vii^	77.283 (2)	Nd^ix^—Se—Nd	107.535 (2)
Se^iv^—Nd—Se^vii^	130.810 (11)	Nd^x^—Se—Nd	162.372 (6)
Se^v^—Nd—Se^vii^	135.510 (1)	Nd^xi^—Se—Nd	81.565 (16)
Se^vi^—Nd—Se^vii^	135.510 (1)	Nd^xii^—Se—Nd	81.565 (16)
Se^i^—Nd—Se	130.811 (11)		

Symmetry codes: (i) −*x*+1/2, −*y*, *z*+1/2; (ii) *y*+1/4, *x*+1/4, *z*+1/4; (iii) *y*+1/4, −*x*−1/4, −*z*+1/4; (iv) −*x*+1/2, *y*, −*z*; (v) −*y*+3/4, −*x*+1/4, *z*+1/4; (vi) −*y*+3/4, *x*−1/4, −*z*+1/4; (vii) *x*, −*y*, −*z*+1/2; (viii) *y*, −*z*, −*x*+1/2; (ix) −*x*+1/2, −*y*, *z*−1/2; (x) −*y*−1/4, *x*−1/4, −*z*+1/4; (xi) *y*, *z*, *x*; (xii) *y*+1/4, −*x*+3/4, −*z*+1/4.
